# Oncolytic Viruses: Newest Frontier for Cancer Immunotherapy

**DOI:** 10.3390/cancers13215452

**Published:** 2021-10-29

**Authors:** Masmudur M. Rahman, Grant McFadden

**Affiliations:** Center for Immunotherapy, Vaccines and Virotherapy, Biodesign Institute, Arizona State University, Tempe, AZ 85287, USA; grantmcf@asu.edu

**Keywords:** oncolytic virus, immunotherapy, cancer therapy, combination therapy, oncolytic, virotherapy

## Abstract

**Simple Summary:**

Oncolytic viruses (OVs) are viruses that selectively target and kill cancer cells while sparing normal ones. OVs are from diverse families of viruses, but naturally occurring OVs have been genetically engineered due to their limitations in therapeutic application. These engineered OVs with enhanced tumor targeting ability, oncolytic activity, or generating potent anti-tumor immune responses are tested in preclinical animal models and cancer patients in clinical trials. Due to their multi-mechanistic anti-tumor effects, OVs have emerged one of the key cancer immunotherapy agents. However, due to the limited success with novel anti-cancer therapies such as immunotherapies and cell-based therapies, combination therapies should be tested with OVs. We discuss such combination therapies that are explored to further improve oncolytic virotherapy.

**Abstract:**

Cancer remains a leading cause of death worldwide. Despite many signs of progress, currently available cancer treatments often do not provide desired outcomes for too many cancers. Therefore, newer and more effective therapeutic approaches are needed. Oncolytic viruses (OVs) have emerged as a novel cancer treatment modality, which selectively targets and kills cancer cells while sparing normal ones. In the past several decades, many different OV candidates have been developed and tested in both laboratory settings as well as in cancer patient clinical trials. Many approaches have been taken to overcome the limitations of OVs, including engineering OVs to selectively activate anti-tumor immune responses. However, newer approaches like the combination of OVs with current immunotherapies to convert “immune-cold” tumors to “immune-hot” will almost certainly improve the potency of OVs. Here, we discuss strategies that are explored to further improve oncolytic virotherapy.

## 1. Oncolytic Virus: Multi-Mechanistic Cancer Therapeutics

### 1.1. Oncolytic Virus: Brief Background and History

Oncolytic viruses (OVs) were selected for development because they can selectively infect and kill cancer cells but spare their normal cellular counterparts. Treatment of cancer with live oncotropic viruses has a long history. Even before the first reported formal clinical trial with an OV in 1949, there were case reports since the mid-1800s suggesting that natural microbial infections in cancer patients can sometimes temporarily regress tumor burden. The potential therapeutic role of viruses in particular was further established in the late 1890s by an observation that a “flu-like” disease associated with diffuse inflammation coincided with reducing tumor cells in a leukemic patient. Beginning 1949, many clinical trials were undertaken using different types of wild-type non-attenuated viruses [1,2,3]. Shortly thereafter, the trend in the OV field evolved to exploiting genetically modified viruses with less pathogenicity to humans, such as live attenuated vaccines. In the past 20–30 years, the transition has continued to the modern era of using genetically modified viruses for cancer therapy, including the exploitation of knockouts of virus genes and/or knockins of therapeutic transgenes, mainly due to enhanced knowledge and tools of engineering viruses and better understanding of the biology of different candidate oncolytic viruses [4,5].

The OV field gained considerable attention after positive results from many clinical trials. So far, four OVs have been approved globally (Table 1). The first OV, a picornavirus called Rigvir, was approved in Latvia to treat melanoma but never achieved widespread use [6]. Secondly, an engineered adenovirus designated H101, was approved in China in 2005 to treat head and neck cancer [7]. Thirdly, in 2015 another OV, an engineered Herpes simplex virus (HSV-1), named Talimogene Laherparepvec (T-VEC), was approved in the USA and Europe for the treatment of non-resectable metastatic melanoma [8]. Finally, in 2021 a modified herpes simplex virus, named DELYTACT was approved in Japan for brain cancers such as glioblastoma [9]. Oncolytic virotherapy received even more attention after realizing that the true potential of viruses in cancer therapy lies in the ability to trigger novel cancer-specific acquired immune responses against tumor antigens. These observations have shifted the application of OVs from purely lytic agents to antitumor immune-activating agents, and the field could now be more correctly called “oncolytic immunotherapy”. Another newer aspect of OV is their potential application in combination therapy with traditional and modern cancer treatment modalities, particularly with immune checkpoint inhibitors (ICIs) and T cell-based therapies.

### 1.2. Types of Viruses in Use/Development as Oncolytic Therapeutics

An ideal oncolytic virus candidate should possess several hallmarks, such as a solid fundamental understanding of their biology and genetics. The OV should be pro-immunogenic, exert lytic activity in the infected malignant cells, should not lead to a chronic or infectious disease, or be capable of integrating into the human genome. Additionally, the virus must be broadly safe for a diverse human population. It is also feasible to genetically modify and arm with recombinant transgenes to enhance its immunogenicity or stimulate targeted anti-cancer mechanisms. Since the early clinical trials, diverse types of viruses with and without genetic alterations were tested and entered into clinical trials. They include members of both DNA and RNA viruses (Table 2). Examples of oncolytic DNA viruses are adenoviruses, herpes simplex virus (HSV), parvoviruses, and poxviruses such as vaccinia virus (VACV) and myxoma virus (MYXV). Examples of oncolytic RNA viruses include Coxsackie virus, Maraba virus, measles virus (MV), Newcastle disease virus (NDV), poliovirus, reovirus, retroviruses, Seneca Valley virus (SVV), Semliki Forest virus (SFV), Vesicular stomatitis virus (VSV), and Sindbis virus (SBV). Oncolytic DNA viruses have the advantages of high genome stability and larger transgenes insertion capability without compromising viral infection and replication [10,11]. On the other hand, RNA viruses have limited genome packaging capacity, but some can be more immunogenic [12]. However, there are pros and cons with every virus that has been developed as OV and tested so far.

## 2. Mechanisms of Cancer Cell Tropism of OVs

### 2.1. Overexpression of Receptor Molecules on the Cancer Cell Surface

Oncotropism of OVs generally depends on multiple factors like cell surface receptors necessary for virus binding/entry (for some, but not, all OVs), cellular metabolic status, and the ability of the virus to overcome intracellular innate immune or antiviral signaling pathways within cancer cells (likely applicable for all OVs). The early observations that some OVs exploit unique extracellular molecules expressed on cancer cells for binding and entry led to this field’s initial growth. For example, CD46, CD155, and integrin α2β1 molecules are frequently overexpressed in many classes of tumor cells, and can serve as the receptor for measles virus, poliovirus, and echovirus, respectively [13,14,15]. However, the same OV might use a different cell surface molecule for different cancer types. For example, measles virus uses CD46 overexpression on multiple myeloma cancer cells while nectin-4 is the major virus receptor for pancreatic, colorectal, breast, and colon carcinomas [16,17]. Other molecules that have a role in tumor growth and progressions, such as anthrax toxin receptor 1 (ANTXR1), lamin receptor, intracellular adhesion molecule-1 (ICAM-1), and decay-accelerating factor (DAF), also can serve as a receptor for SVV-001, Sindbis virus, and coxsackievirus, respectively. Oncolytic HSV infection of cancer cells relies on increased expression of multiple receptors in cancer cells such as herpesvirus entry mediator (HVEM), a member of the TNF superfamily, and nectin-1 [18]. Members of oncolytic poxviruses such as VACV and MYXV displayed natural cancer cell tropism and selectively targeted tumors but this specificity is mainly because virus binding and entry is not mediated by selective receptor molecules on the cell surface (and thus virus binding is relatively promiscuous for both normal and cancerous cells), but rather is determined by the innate intracellular environment in cancerous cells being less inhibitory to the virus than in normal primary cells [19,20].

### 2.2. Alteration in Intracellular Signaling Pathways

Cancer is a complex, heterogeneous disease with multiple genetic mutations that mediate frequent compromises in the various antivirus signaling pathways, which serve as a perfect niche for OV replication. Different OVs exploit selective defects in cancer cells, understanding of which remains an active area of research. In general, cancer cells during the transformation process selectively sacrifice at least some elements of their potent innate antiviral response pathways mediated by cytokines like type I and II interferons (IFNs) or tumor necrosis factor (TNF) [21]. Although compromise in cytokine-mediated induction of the antiviral state is the basis for many OVs’ abilities to infect cancer cells and not their normal counterparts, there are also multiple other innate defense pathways that sense and block viral replication and are key for OVs’ ability to selectively infect and replicate in cancer cells. For example, cancer-specific aberrations in *RAS*, *TP53*, *RB1*, *PTEN*, *EGFR*, *WNT*, *BCL-2*, and other cancer-related genes can often further predispose cancer cells to viral infection [20,22,23,24,25]. In a heterogeneous tumor microenvironment, there are possibly more mutations that are yet to be identified in both cancerous and non-transformed support cells that likely affect virus tropism as well.

### 2.3. Altered Metabolism of Cancer Cells

Most tumor cell types are characterized by a high rate of aerobic glycolysis (Warburg effect), which plays a vital role in developing the immunosuppressive tumor microenvironment (TME) [26]. This is partly because tumor cells cause excessive depletion of extracellular glucose, which in turn restricts glucose availability to resident immune leukocytes, which eventually reduce proliferation and effector function of immune cells such as tumor-resident T cells. In addition, accumulation of tissue lactate due to increased glycolysis in the TME also severely impacts the functional properties of local T cells and NK cells [27,28]. Studies have shown that inhibition of tumor cell glycolytic metabolism enhanced antitumor immune responses and the function of chemotherapy drugs [29,30]. Viruses upon infection of host cells also tend to activate glycolysis, enhancing the synthesis of cellular biomolecules and viral particles, thus amplifying the Warburg effect. Viruses explore diverse mechanisms for enhancing glycolysis, as a strategy to favor virus replication, but many of the details remain to be fully described [31]. Different drug inhibitors have been identified that can reduce cancer cell metabolism, but they also can function as antiviral drugs and some might reduce the therapeutic benefits of OVs if used concurrently. More studies are needed to establish the synergistic impacts of OV vs. metabolic inhibitors. However, some studies suggest that selected inhibitors might have no effect or even enhance OV replication and, at the same time, target cancer cell metabolism. For example, upregulation and activation of hexokinase 2 (HK2), the first rate-limiting enzyme of glycolysis, is a key event for glycolysis. Inhibition of HK2 with lonidamine enhanced oncolytic alphavirus M1 replication [32]. OV treatment together with glycolysis inhibitor can enhance cancer cell death. For example, inhibition of HK with D-Mannoheptulose, a specific hexokinase inhibitor, together with NDV resulted in inhibition of glycolysis and enhanced apoptotic cancer cell death [33]. NDV-infected breast cancer cell lines showed a decrease in the hexokinase (HK) activity, pyruvate and ATP concentrations, and acidity, all of which reflect a significant decrease in the glycolytic activity resulting in induction of apoptosis in cancer cells but not in normal cells [34]. Similarly, dichloroacetate (DCA), an inhibitor of glycolysis, enhanced oncolytic measles virus replication and promoted necrotic cell death [35].

## 3. Mechanisms of Antitumor Effects Mediated by OVs

After binding and entering tumor cells, OVs can exploit multiple lytic mechanisms to kill the infected cancer cells that may or may not be linked to the actual extent of virus replication within the target cells. The exact mechanisms of viral oncolysis are still incompletely understood, and vary widely from virus to virus, and can even differ dramatically between diverse target cancer cell types. OVs are thought to mediate antitumor activity through multiple mechanisms: (a) selective virus replication within cancer cells, causing direct cytolytic effects (a mechanism also known as oncolysis) [36,37,38]; (b) indirect effects of cell death (e.g., apoptosis-like vs. necrosis-like) on both infected and uninfected cancer cells and associated endothelial cells in the tumor-associate vasculature leading to reduced angiogenesis [39,40]; and (c) activation of systemic antitumor (and antiviral) immunity and recruitment of activated immune cells into the TME [41,42,43,44]. However, these mechanisms differ widely from virus to virus, the nature and type of cancer cells, and the overall interaction among the OV, TME, and host immune system. Most viruses antagonize the host-induced cell death pathways that get activated upon virus infection. In some cases, virus-encoded proteins are known to target different types of cell death pathways, either as inhibitors or inducers [45,46]. However, once infected by an OV, the cancer cells will usually die from the induction of cell death pathways and/or cell integrity failure caused by virus-induced cell damage. Additionally, for preferential induction of cell lysis, some OVs have been engineered to specifically activate different types of cancer cell death pathways such as apoptosis, necrosis, autophagy, or pyroptosis. The term immunogenic cell death (ICD) is usually used to describe the kind of cancer cell death that can expose cancer cell antigens to the resident immune cells in the TME and is often measured in cultured cells by extracellular exposure of normally intracellular markers or the cell release of intracellular mediators. The advantages of OVs are that they can trigger multi-mechanistic cell death pathways within the tumor bed. Among these, ICD is believed to play a crucial role in promoting acquired anti-tumor immunity [47,48]. When the replication of OVs in cancer cells induces ICD, this results in the release of tumor-associated antigens (TAAs), damage-associated molecular patterns (DAMPs), OV-derived pathogen-associated molecular patterns (PAMPs), and upregulation of multiple inflammatory cytokines, all of which subsequently activate both innate and adaptive immune responses. The release of DAMPs such as extracellular ATP and high mobility group box 1 (HMGB1) proteins and those cytoplasmic proteins that become exposed at the cell surface, such as HSP (Heat shock protein) 70, HSP90, and calreticulin (CRT) are all hallmarks of ICD. After secretion, DAMP molecules bind to their receptors CD91 (CRT), P2RX7 (ATP), and TLR4 (HMGB1) on dendritic cells (DCs), which subsequently mature, process antigens, and then educate/activate T cells to enhance antitumor responses [49,50]. Extracellular ATP and surface-exposed CRT act as ‘find me’ and ‘eat me’ signals to phagocytic immune cells. At the molecular level, cGAS, a DNA sensor that responds to cell stress by binding to abnormal cytoplasmic DNA in infected cells and activate STING pathways, trigger innate immunity using type I IFN gene expression, the release of chemokines CXCL9 and CXCL10, and ultimately the recruitment of T cells [51,52]. With oncolytic virotherapy, ICD is particularly important for development of antitumor immunity at metastatic sites. Recent studies have shown that OVs including adenovirus, parvovirus, reovirus, coxsackievirus, VACV, NDV, and HSV all induce varying degrees of ICD. OV-mediated induction of ICD plays a crucial role in converting lymphoid-deficient or low immune sensor expressing tumors (i.e., “cold” tumors) into T cell-inflamed tumors (i.e., “hot” tumors) [53,54,55]. Apart from ICD, autophagy also can induce antitumor immune responses due to OV infection and replication in cancer cells. For example, induction of autophagy enhanced replication of oncolytic Adenoviruses and NDV [56,57]. Autophagy also enhanced antitumor effects via oncolysis, autophagic cell death, and ICD [58,59,60,61].

## 4. Challenges/Limitations with OV to Become Successful as Monotherapy

Like many other modern cancer therapies, there are still challenges and obstacles ahead with oncolytic virotherapy and becoming a successful anticancer therapy. Some of the key factors that contribute to the limitation of OV functions are: (1) unknown host antiviral pathways that limit the OV activity and spread in the tumor bed, (2) surrounding intrinsic physical barriers in the tumor bed limiting OV access, and (3) adaptive immune responses limiting viral functions indirectly. Furthermore, there are additional factors that should be considered:

- Selection of optimal OV candidate: Until now, multiple DNA and RNA viruses have been explored as OV candidates. To be an ideal candidate, there are various properties that the selected virus should have, such as the ability to incorporate transgenes stably, little or no toxicity to normal cells and tissues, immunogenicity, large scale clinical grade amplification, production optimization and appropriate therapeutic targets of the chosen OVs.
- Virus entry, infection, and spread: OVs that use selected cell surface receptors for binding and entry are often not useful for tumors with reduced or no expression of those receptors. Although this barrier has been overcome for some viruses by engineering, a few viruses (e.g., poxviruses) can circumvent this issue by binding to nonspecific determinants like ubiquitously expressed cell surface glycosaminiglycans. At the intracellular level, there are additional complex signaling pathways that are directly or indirectly linked with the antiviral pathways and commonly restrict virus replication (if operative) and spread to the new cells. For example, AKT activation levels regulate MYXV replication in human cancer cells [62,63]. In the tumor bed, the presence of excessive extracellular matrix (ECM) can prevent viral spread. For example, fibrillar collagen in the ECM limits oncolytic HSV spread within tumors [64].
- Delivery of OV: Delivery of OV to the sites of primary and metastatic sites is vital for optimal therapeutic outcomes. In this regard, since only a minority of human cancers are amenable to direct intratumoral (IT) injection, systemic delivery is the preferred route compared to IT injection of the virus. However, there are several barriers to the successful delivery of any OV. The presence of neutralizing antiviral antibodies, complement activation, expression of antiviral cytokines, and natural clearance site of OVs by the liver and spleen are all major obstacles in the systemic OV delivery. Although IT delivery of virus can circumvent some of these barriers, tumor beds can limit virus spread, and the tumor vasculature is also a limiting factor to IT and metastatic sites. One strategy to overcome these issues is to exploit migratory leukocytes as carrier cells to ferry the virus into tumor beds that allow cellular ingress.
- Neutralizing antibodies and antiviral cytokines: Preexisting neutralizing antiviral antibodies are the main obstacle in the context of systemic delivery of free virus to reach the tumor bed [65]. Additionally, the host immune system activates antiviral immunity and limits the oncolytic activity of OVs. The virus-sensing cellular receptor molecules that detect virus particles and virus-infected cells activate type I IFN signaling pathways, which activates antiviral defense pathways in the uninfected cells and limits OV infection and spread. Moreover, the immune clearance of infected cells, including cancerous cells, prevents virus spread although it can be an important feature of activating antitumor immune responses.
- Immunosuppressive TME: Another barrier to OV therapy is the frequent presence of highly immunosuppressive TME. In the tumor bed, various cellular subsets like cancer cells, stromal cells, inhibitory cytokines (e.g., TGF-beta) and infiltrating immune cells (e.g., regulatory T cells and myeloid derived suppressor cells) all contribute to the immunosuppressive TME. Although this is critical for the tumor to evade the host’s innate and adaptive immunological defenses, OVs must function within this immunosuppressive TME. Additionally, some OV infections can further promote the tumor bed’s immunosuppressive environment by activating the immune system. For example, Maraba virus upregulated the PD-1/PD-L1 axis on tumor cells and tumor-infiltrating immune cells [66,67]. Similarly, oncolytic NDV also promoted PD-L1 production in the tumor bed in response to the virus stimulated type I IFN signaling, resulting in an immunosuppressive TME even in distant tumors [68,69].

## 5. Engineered Oncolytic Viruses

The genetic engineering of OVs has now become an integral part of developing safe, cancer-selective, and highly effective OVs against diverse types of cancers. Engineered OVs have overcome some of the challenges that are listed in the above section. Any modification of OV relies heavily on understanding the biology and genetic information of the virus, virus–host interactions, how viruses kill infected cells, and how cells protect themselves from the lytic infection. Genetic engineering by knockout deletion of certain viral genes can enhance OV tumor cells tropism and reduced toxicity for normal cells; engineering and arming via knockins with different ectopic transgenes has enabled OV application as oncolytic immunotherapy to more broadly activate the anti-tumor immune responses. This field of developing engineered OVs and arming OVs with transgenes is rapidly expanding due to the recent discovery of many new biologics with diverse potential as immunotherapy. During the past few years, many reviews have been written on this topic and we have briefly highlighted some of the key engineering of OVs that substantially improved application of OV as cancer therapeutics [70,71,72,73].

Based on the purpose and type of transgenes used for OV engineering and modifications, they can be classified into many groups. Some examples are listed here: (i) deletion/mutation of viral genes for selective replication in tumors and protecting normal tissues. For example, ICP34.5 and ICP47 genes were deleted in T-VEC [8]. ICP34.5 protein encoded by HSV-1 is responsible for neurovirulence and required for inhibition of interferon response, pathway which is frequently defective in tumor cells but active in normal cells. Thus, the ICP34.5 deleted virus can selectively replicate in cancer cells and safe in the brain [74,75]. (ii) Substitution/insertion of proteins from other viruses for tumor targeting. For example, VSV glycoprotein G has been substituted with a glycoprotein variant of lymphocytic choriomeningitis virus (LCMV) for selective replication in cancer cells [76]. Similarly, mutation or deletion in the thymidine kinase (TK) gene in HSV-1 and VACV and deleting E1B55K in oncolytic adenovirus ONYX-015 allowed selective replication in cancer cells [77,78]. (iii) Arming OVs with immunostimulatory cytokines and chemokines. OVs expressing cytokines like GM-CSF, TNF, IFN-a/b, IL-2, IL-7, IL-12, IL-15, IL-18, IL23, IL-24, and FLT3L have shown enhanced anti-tumor immune responses and tumor reduction in multiple preclinical cancer models and clinical trials [79]. Expression of these cytokines in the tumor bed using OVs greatly reduced the toxicity associated with their systemic delivery to patients. Early success with T-VEC expressing GM-CSF, which enhanced DC and APC recruitment to the tumor sites, allowed expression of other cytokines using OVs for modulation of immunosuppressive TME. IL-12 expression using different OVs has shown potent antitumor activity in preclinical studies [80,81,82]. Currently, HSV (M032) and VACV (ASP9801) expressing IL-12 are in clinical trials (Table 3). Chemokines such as CCL2, CCL5, CLL19, CCL20, CCL21, and CXCL11 have been expressed using OVs to enhance the migration of immune cells into TME [79]. Oncolytic adenovirus (NG641) expressing CXCL9, CXCL10, and IFNα is in clinical trial (Table 3). Other OVs expressing different chemokines showed improved efficacy in preclinical cancer models [83,84]. (iv) Expression of immune-activating ligands. Immune-activating ligands such as TRAIL, CD40L, OX40L, 4-1BBL, B7-1, and GITR expression using OVs has shown promising results in preclinical models and in clinical trials. For example, oncolytic adenovirus (LOAd703) expressing CD40L and 4-1BBL has shown immune activation in different cancer models and currently in clinical trial [85,86]. (v) Expression of immune checkpoint inhibitors. OVs have been engineered to directly express checkpoint blockade antibodies such as anti-PD1, anti-PDL1, or anti-CTLA4 in the tumor bed [87,88,89,90,91]. This is mainly to overcome the toxicity associated with systemic delivery of ICIs. For example, expression of anti-PD-1 mAb in HSV-1 enhanced anti-tumor immune responses and T-cell infiltration in TME [91]. (vi) Arming OVs with bispecific immune cell engagers. OVs expressing antibodies targeting tumor antigens and capable of activating T cell receptor signaling, such as Bi- or tri-specific T cell engager (BiTE or TriTE), are tested for targeted immunotherapy [92]. A BiTE and TriTE-armed oncolytic Adenovirus showed depletion of tumor associated macrophages in cancer patient samples [93]. However, the success with these immune-modulators will depend on the identification of tumor associated antigens. (vii) Activation of immunogenic cell death. OVs have been engineered to express pro-death molecules such as beclin-1 in enhancing ICD and autophagic cell death [94,95].

## 6. Combination Therapy with Oncolytic Virus

**Figure 1:**
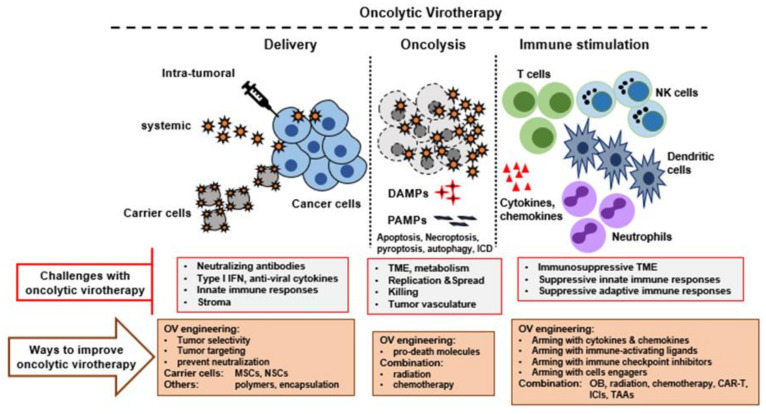
Therapeutic features of oncolytic virotherapy and ways to improve it. Multiple steps are involved in successful oncolytic virotherapy. First, the virus must be delivered successfully to the tumor bed or TME using an optimized delivery method. Second, the OV must replicate and spread efficiently in the tumor bed, causing oncolysis and release of tumor selective immune stimulating molecules. Third, the OV must function as an immunotherapeutic agent to activate strong innate and adaptive anti-tumor immune responses. All these steps can be further improved by engineering OVs to express suitable transgenes and by combination with other agents or therapies. TME, tumor microenvironment; DAMPS, danger associated molecular patterns; PAMPs, pathogen associated molecular patterns; ICD, immunological cell death; MSCs, Mesenchymal stem cells; NSCs, neural stem cells; ICIs, immune checkpoint inhibitors; TAAs, tumor associated antigens.

OVs provide multi-mechanistic therapeutic effects against most types of cancers (Figure 1). However, in clinical trials of monotherapy, OVs with earlier generations of armings (such as GM-CSF) have shown complete response in relatively few patients. Although engineering of OVs with different approaches enhanced oncolytic activity and activated the antitumor immune responses, better therapeutic outcomes were reported when oncolytic viruses were used in combination with other cancer treatment modalities, such as chemotherapy, radiation therapy, immunotherapy, or cell therapy [96,97,98]. Examples of currently active clinical trials with OV and different combination therapies are presented in Table 3.

### 6.1. Combination with Traditional Radiotherapy and Chemotherapy

Traditional therapies such as radiotherapy (RT) and chemotherapy have been used either alone or in combination. Radiotherapy is mostly used for the local control of tumors and displays a wide range of antitumor effects [99]. However, due to OVs limited success, radiotherapy plus OVs have been studied as a combination therapy in preclinical models and a limited number of clinical trials. Oncolytic VACV, HSV, VSV, and adenovirus have shown therapeutic benefits combined with RT [100,101,102]. The combination of RT with OV has synergistic antitumor effects, and can be particularly effective against aggressive tumors for which other therapies failed [103]. For example, OV Delta-24-RGD in combination with RT was tested in pediatric high-grade gliomas (pHGG) and diffuse intrinsic pontine gliomas (DIPGs) models [104]. In these models, OV downregulated DNA damage repair proteins, sensitized tumor cells to the effect of RT, enhanced trafficking of immune cells, and enhanced overall survival of mice. Thus, OV-mediated inhibition of cellular DNA repair pathways can sensitize tumors with RT [102]. Similarly, oncolytic VSV expressing IFNβ (VSV-IFNβ) in combination with RT enhanced antitumor immune response and tumor reduction in syngeneic models [105]. Currently, a phase I clinical trial for locally advanced rectal cancer with a chimeric adenovirus type 11p (Enadenotucirev) and chemoradiotherapy, radiotherapy, and chemotherapy (capecitabine, a non-cytotoxic pre-cursor of 5-fluorouracil) is in progress (NCT03916510).

Conventional chemotherapeutics were tested with OVs to enhance the therapeutic effects of OVs. The goal was to reduce the dosage and toxic effects of the drug while enhancing the efficacy of OVs in the tumor microenvironment. However, in some cases, depending on the type of chemotherapeutic drugs and dose regimens used, certain drugs acted as antivirals and also reduced viral replication in the tumor bed. This combination therapy was tested in many preclinical tumor models [106,107]. The positive results from these early studies led to the start of clinical trials on several types of aggressive tumors such as the brain, pancreatic, breast, melanoma, ovarian, and myeloma using OVs with standard chemotherapeutics drugs, for example, cisplatin, paclitaxel, doxorubicin, gemcitabine, temozolomide, cyclophosphamide, and doxycycline. In recent reviews, these combinatorial studies are highlighted in greater detail [96].

### 6.2. Systemic Delivery of OV via Carrier Cells

Unlike intratumoral delivery, systemic delivery of OV faces many challenges, mainly due to the highly efficient immune filtration system that detects and removes pathogens from the circulatory system. Many types of carrier cells have been tested to determine their suitability as a delivery vehicle for systemic administration of OVs to overcome the deficits of intravenously infusing naked virus. In preclinical studies, multiple OVs have been tested with different carrier cell types demonstrating the success and feasibility of this approach and potential clinical application [108,109]. This is particularly evident for viruses like measles virus, reovirus, and many other viruses that encounter substantial levels of preexisting antibodies. Among the many carrier cells tested to date, patient-derived mesenchymal stem cells (MSCs) have gained attention and have been tested in clinical trials with OVs. MSCs with measles virus have shown promise and delivery to the tumor bed in clinical trials (NCT02068749). OV-infected carrier cells have demonstrated enhanced migratory properties and secretion of proinflammatory cytokines, for example, human umbilical cord MSCs, suggesting that they can enhance the antitumor efficacy of virotherapy [110]. Apart from MSCs, neural stem cells (NSCs) also improved the delivery of OV in multiple cancer models [111,112,113]. Another potential carrier cell is represented by chimeric antigen receptor T (CAR-T) cells that have been engineered to recognize and kill target cancer cells. Tumor-infiltrating T cells also have excellent potential, if sufficient quantities can be isolated from patients. Since any migratory leukocyte can in theory have potential as a carrier cell for at least some OVs, the future for this line of inquiry looks bright.

### 6.3. Combination with Immune Checkpoint Inhibitors (ICIs)

ICIs that target immune checkpoints like PD-1, PD-L1, or CTLA-4 and disrupt the cancer cell’s ability to evade the host immune response have become one of the key cancer therapy modalities. However, despite many success, there are several critical limitations of ICIs: (i) even with optimally targeted cancers, only limited fraction (10–20 percent) of patients respond to ICI treatment, (ii) immune-related adverse effects reported in some patients receiving ICI therapy, and (iii) Limited effect against immunologically “cold” tumors characterized by a low tumor-infiltrating lymphocyte (TILs). In this context, OVs, particularly genetically engineered OVs, can enhance lymphocyte infiltration and activation as well as lysis of cancer cells. Thus, to overcome this lack of ICI effectiveness in too many patients, OV virotherapy combined with ICIs has been tested in preclinical models and clinical trials, and has shown promising results [114,115,116,117] OVs that are currently under clinical trials in combination with ICIs are HSV (OH2), VACV (PexaVec), Reovirus (Reolysin), Adenovirus (LOAd703), NDV (MEDI5395), and VSV (VSV-IFNb-NIS) (Table 3). However, recent studies suggest that genetically engineered OVs and OVs expressing immune stimulating cytokines that have greater potential of altering TME have shown enhanced efficacy in combination with ICIs. For example, a live attenuated ZIKV vaccine candidate and a recombinant orthopoxvirus (CF33) oncolytic activity was enhanced by immunotherapy [118,119]. Oncolytic HSV-1 (ONCR-177), adenovirus (TILT-123), and MYXV (vMyx-hTNF) expressing single or multiple immune stimulatory cytokines enhanced oncolytic activity when combined with ICIs [120,121,122].

### 6.4. Combination with Cell Therapy

Cellular immunotherapy, also known as adoptive cell therapy, uses modified versions of the cells of the immune system to eliminate cancer cells. Various types of cellular immunotherapies have been developed: such as chimeric antigen receptor (CAR) T cells therapy, engineered T cell receptor (TCR) therapy, tumor-infiltrating lymphocyte (TIL) therapy, and natural killer (NK) cell therapy. Among these cell-based therapies, CAR-T cell therapy has shown remarkable efficacy for blood cancer as emerging immunotherapy. However, cell therapy has also a limited success, particularly in solid tumors, due to the lack of infiltration and prolonged existence in the tumor tissue. To overcome these limitations, cell therapy has been tested in combination with OVs. In this case, the use of engineered OVs expressing therapeutic transgenes can enhance the therapeutic benefit of both treatment strategies. For example, oncolytic adenovirus expressing IL-7, when combined with B7H3-targeted CAR-T, showed higher efficacy than a single use of either of those treatments [123]. Combination with OV enhanced T cell proliferation and reduced T cell apoptosis. Cell therapy can be further explored in combination with multi-armed OV. For example, an adenovirus-based OV expressing cytokine, checkpoint blockade, and a BiTE molecule was used and HER2-specific CAR T cells significantly improved tumor control and survival [124].

## 7. Conclusions

More than three decades of extensive research and clinical trials have established oncolytic virotherapy as a promising treatment modality for cancer treatment. Several aspects of OV therapy have been significantly improved, including safety, potency, selectivity, delivery methods, and production. Perhaps the most notable shift in the OV field has been from its application as a direct lytic agent to development as a multimodal agent involving cell lysis, immune stimulation, and gene therapy, which further established OV as a strong candidate for cancer therapy. However, it is becoming clear that OVs as a single anti-cancer agent might not successfully provide a complete response for cancer cure, such that combinatorial strategies are essential. Mainly because of the heterogeneous nature of the cancer cells, there are increasing chances of recurrence, metastasis, and failure to diagnose early. Therefore, like other combination therapy, it is possible to exploit OV in combination with existing cancer therapies, an area still less explored. However, in this case, a rational design and combination approach can mitigate the inherent limitations of OVs and other therapies against selected cancer types. For example, armed OVs with enhanced tumor specific replication ability and stimulating a potent anti-tumor immune response can be combined with immune checkpoint inhibitors and cell therapies for cancer that develop resistance against current therapies because of rapid mutations and heterogeneous cell populations.

## Figures and Tables

**Table 1:** List of approved oncolytic viruses.

Name	Virus Type	Host	Year Approved	Country Approved	Indication	Background
Rigvir (ECHO-7)	Picornavirus	Human	2004	Latvia	Melanoma	Unmodified
Oncorine (H101)	Adenovirus serotype 5	Human	2005	China	Head and neck cancer	Deleted for viral E1B-55K and with four deletions in viral E3
T-VEC (Imlygic)	HSV-1	Human	2015	United States and Europe	Metastatic melanoma	Deletion of ICP34.5 and ICP47; encoding two copies of human GMCSF
DELYTACT (teserpaturev/G47Δ)	HSV-1	Human	2021	Japan	Malignant glioma or any primary brain cancer	Triple mutation (Deletion of ICP34.5, ICP6 and α47 genes)

**Table 2:** List of selected oncolytic viruses.

Virus	Genome (Size)	Cell Receptor/Binding Determinants	Replication Site	Vertebrate Host	Examples of OV Candidates
**DNA Virus:**					
Adenovirus	dsDNA (35 kb)	CAR	Nucleus	Human, animals	DNX-2401, ONCOS-102, AD-E6E7
Herpesvirus: HSV-1, HSV-2	dsDNA (154 kb)	HVEM, Nectin 1, Nectin 2	Nucleus	Human (HSV-1)	T-VEC, OH2, HSVG207, M032
Parvovirus: B19PV, H1PV	ssDNA (5 kb)	Sialic acid residues, P antigens	Nucleus	Human, animals	ParvOryx01
Poxvirus: VACV, MYXV	dsDNA (160–190 kb)	Heparan, laminin, chondroitin, integrin β1, CD98	Cytoplasm	VACV (unknown), MYXV (rabbit)	Pexa-Vec, JX-594
**RNA Virus:**					
Alphavirus: Semiliki Forest virus (SFV), Sindbis virus (SINV), M1	SS (+) RNA (11–12 kb)	Prohibitin, phosphatidyl serine, GAGs, ATP synthetase β subunit	Cytoplasm	SFV: rodents/human; SINV: birds	SFV-IL12; SINV AR339
Flavivirus: Zika virus	SS (+) RNA (10.8 kb)	GAGS, Heparan sulfate, C-type lectin	Cytoplasm	Monkey	ZIKV-LAV
Paramyxo virus:					
Measles virus	SS (−) RNA (16 kb)	SLAMF1 (CD150), CD46, Nectin 4	Nucleus	Human	MV-NIS
Newcastle disease virus (NDV)	SS (−) RNA (15 kb)	Sialic acid	Nucleus	Birds	MEDI5395
Picornavirus:					
Coxsackievirus A21	SS (+) RNA (28 kb)	CAR, ICAM-1, DAF	Cytoplasm	Human	CVA21, CV-B3
Polio virus	SS (+) RNA (7.5 kb)	CD155	Cytoplasm	Human	PVSRIPO
Seneca valley virus (SVV)	SS (+) RNA (7 kb)	Anthrax toxin receptor 1	Cytoplasm	Pig, cow	SVV-001
Reovirus	dsRNA (23 kb)	Sialic acid, JAM1	Cytoplasm	Human	Reolysin
Rhabdovirus:					
VSV	SS (−) RNA (11 kb)	LDLR	Cytoplasm	Cattle, horse, pigs	VSV-IFNβ-NIS
Maraba virus: MG1	SS (−) RNA (11 kb)	LDLR	Cytoplasm	Amazonian phlebotomine sand flies	MG1MA3

**Table 3:** List of selected oncolytic viruses currently in clinical trials.

Virus	Biological Agent	Genetic Modifications/Transgenes	Combination Therapy	Indication (Delivery Route)	Clinical Phase	Clinical Trial No
**Adenovirus**	LOAd703	TMZ-CD40L 4-1 BBL	Gemcitabine, nab-paclitaxel, +/− anti-PD-L1	Pancreatic cancer (IT)	I/II	NCT02705196
	DNX-2440			Recurrent glioblastoma (stereo tactically)	I	NCT03714334
	TILT-123	TNF, IL-2		Solid tumor (IT)	I	NCT04695327
	TILT-123	TNF, IL-2	Adoptive cell therapy with TILs	Metastatic melanoma (IT/IV)	I	NCT04217473
	Enadenotucirev (Ad3)		Capecitabine, radiotherapy	Locally advanced rectal cancer (IT)	I	NCT03916510
	NG-641	FAP-TAc antibody + CXCL9/CXCL10/IFNa		Metastatic cancer, epithelial tumors (IT/IV)	I	NCT04053283
	AdAPT-001			Solid tumor (IT)	I	NCT04673942
	Ad5 OBP-301 (Telomelysin)	hTERT		Melanoma stage III/IV (IT)	II	NCT03190824
**HSV**	OH2 (HSV2)	GMCSF		Advanced or metastatic pancreatic cancer	I/II	NCT04637698
	OH2	GMCSF	HX008 (anti-PD-1)	Advanced solid tumors	I/II	NCT03866525
	OH2	GMCSF	HX008 (anti-PD-1)	melanoma	I/II	NCT04616443
	OH2	GMCSF	Keytruda (anti-PD-1)	Advanced solid tumors, melanoma	I/II	NCT04386967
	HSVG207 (HSV-1)	Tumor selective mutation	Low dose of radiation (5 Gy)	Pediatric brain tumor, recurrent or refractory cerebellar brain tumor	I	NCT03911388
	HSVG207	Tumor selective mutation	Low dose of radiation (5 Gy)	Recurrent high-grade glioma in children	II	NCT04482933
	M032 (NSC733972)	IL-12	Low dose of radiation (5 Gy)	Recurrent malignant glioma	I	NCT02062827
**Parvovirus (H-1 PV)**	ParvOryx			Pancreatic ductal carcinoma	I/II	NCT02653313
**VACV**	Pexa-Vec (JX-594)	GM-CSF (TK inactivated)	Tremelimumab (anti-CTLA4), Durvalumab (anti-PD-L1)	Colorectal cancer	I/II	NCT03206073
	TBio-6517/RIVAL-01		Pembrolizumab (anti-PD-1)	Advanced solid tumors (IT)	I/II	NCT04301011
	T601	TK-RR deletion, expression of FCU1 gene	5-FC	Advanced malignant solid tumors	I/II	NCT04226066
	ASP9801	IL-7, IL-12		Metastatic advanced solid tumors (IT)	I	NCT03954067
**NDV**	MEDI5395	GMCSF	Durvalumab (anti-PD-L1)	Advanced solid tumors (IT)	I	NCT03889275
**Reovirus**	Reolysin		Anti-PD-1	Metastatic TNBC	II	NCT04445844
	Reolysin		Chemotherapy (Dexamethasone, carfilzomib), ICIs (Nivolumab)	Relapsed multiple myeloma	I	NCT03605719
	Reolysin		Chemotherapy (paclitaxel), ICI (anti-PD-L1)	Metastatic breast cancer	II	NCT04215146
	Reolysin		Chemotherapy (letrozole), ICIs (atzolizumab, trastuzumab)	Breast cancer	I	NCT04102618
**VSV**	VSV-IFNβ/TYRP1	IFNβ, tyrosinase related protein 1		Stage III-IV melanoma (IT/IV)	I	NCT03865212
	VSV-IFNβ-NIS	IFNβ, NIS	Avelumab (anti-PD-L1)	Malignant solid tumor (IT)	I	NCT02923466
	VSV-IFNβ-NIS	IFNβ, NIS	Cyclophosphamide, ruxolitinib phosphate	MM, AML, T cell lymphoma (IV)	I	NCT03017820
	VSV-IFNβ-NIS	IFNβ, NIS	Pembrolizumab (anti-PD-1)	Refractory NSCLC, HNSCC, solid tumor (IV)	I	NCT03647163
**Maraba**	MG1MA3 (MG1 Maraba/MAGE-A3)	MAGE-A3		Advanced/metastatic solid tumors	I/II	NCT02285816
